# Micro-costing in health and medicine: a critical appraisal 

**DOI:** 10.1186/s13561-020-00298-5

**Published:** 2021-01-06

**Authors:** Xiao Xu, Christina M. Lazar, Jennifer Prah Ruger

**Affiliations:** 1 Department of Obstetrics, Gynecology and Reproductive Sciences, Yale School of Medicine, New Haven, USA; 2 Department of Psychiatry, VA Connecticut Healthcare System, Yale School of Medicine, New Haven, USA; 3 Department of Medical Ethics and Health Policy, Perelman School of Medicine, The Leonard Davis Institute of Health Economics, School of Social Policy & Practice, University of Pennsylvania, 3701 Locust Walk, Philadelphia, PA 19104 USA

**Keywords:** Micro-costing, Economic evaluation, Resource allocation, Health and medicine

## Abstract

**Background:**

Concerns about rising health care costs require rigorous economic study to inform clinical and policy decision-making. Micro-costing is a cost estimation methodology employing detailed resource utilization and unit cost data to generate precise estimates of economic costs. Micro-costing studies have not been critically appraised.

**Methods:**

Critical appraisal of micro-costing studies in English. Studies fully or predominantly employing micro-costing were appraised for methodological and reporting quality through economic evaluation guidelines (Evers, Drummond, Consolidated Health Economic Evaluation Reporting Standards (CHEERS), Fukuda and Imanaka checklists). Following the Panel on Cost Effectiveness in Health and Medicine, micro-costing studies were defined as involving “direct enumeration and costing out of every input consumed in the treatment of a particular patient.”

**Results:**

Full or predominant micro-costing studies included  neoplasms (18.5%), infectious and parasitic diseases (17.9%), and diseases of circulatory systems (10.8%) as the  most studied diseases. 36.9% were in the United States and 34.9% were in Europe. 33.8% did not report analytic perspective, 32.8% did not report price year, 3.6% did not inflation adjust cost data, and 44.1% did not specify inflation adjustment. 86.2% did not separately report unit costs and resource utilization quantity, 14.9 and 19.5% did not provide sufficient detail to assess appropriateness of measured physical units or valued costs.

**Conclusions:**

Micro-costing studies vary widely in methodological and reporting quality, highlighting the need to standardize methods and reporting of micro-costing studies and develop tools for their evaluation.

**Supplementary Information:**

The online version contains supplementary material available at 10.1186/s13561-020-00298-5.

## Key messages

(1) Micro-costing studies vary widely in methodological and reporting quality; (2) There is a need to standardize methods and reporting of micro-costing studies and develop tools for their evaluation.

## Introduction

Concerns about rising health care costs require rigorous economic study to inform clinical and policy decision-making. Advances in medical knowledge and technology results in rapid diffusion of new health interventions that cannot be precisely costed by existing estimates or prices. Micro-costing is particularly relevant for estimating costs of new interventions, but existing interventions, too, benefit from this methodology. By measuring detailed resource utilization and unit costs, micro-costing methodology generates precise cost estimates and has been considered the preferred method for estimating costs of health interventions [1].

The conduct, appraisal and reporting of micro-costing studies can be complex due to the detailed cost categorization and data collection involved. Existing guidelines for economic evaluations, the Drummond checklist [2], Evers checklist [3], and Consolidated Health Economic Evaluation Reporting Standards (CHEERS) checklist [4], have focused on full economic evaluations. Although these guidelines have been instrumental in enhancing the quality, transparency, and comparability of economic evaluation studies, they do not provide sufficient guidelines regarding appropriate techniques and standards to use in micro-costing studies. Moreover, while the recently convened Second Panel on Cost-Effectiveness in Health and Medicine [5, 6] updated recommendations concerning the conduct of economic evaluations, there remains no standardized requirement about how to conduct, appraise, or report micro-costing studies [7].

Given this gap in the literature, current practices in conducting and reporting micro-costing studies likely vary substantially. This may hinder comparison of research findings across studies and interpretation of study results. The objective of this study was to critically appraise micro-costing studies to assess their methodological and reporting quality and the appropriateness of existing economic evaluation guidelines for this purpose.

## Methods

A critical appraisal of micro-costing studies in English was conducted. Ovid MEDLINE, EconLit, BIOSIS Previews, Embase, Scopus, and National Health Service Economic Evaluation Database were searched for records through July 2015. Following Gold and colleagues [1], micro-costing studies were defined as those that involved “direct enumeration and costing out of every input consumed in the treatment of a particular patient”. To inform future design and reporting of micro-costing studies, analysis was limited to studies that fully or predominantly used micro-costing in their cost estimation and studies that only partially or minimally involved micro-costing were excluded. To inform future research, the focus was on research studies with a defined patient sample and research question, studies with a primary goal to inform hospital financial management or estimate unit cost per medical service were excluded.

A detailed data collection form was developed to extract comprehensive data on study design and reporting characteristics for each micro-costing study. Data collected included study population, study intervention, analytical perspective, type of economic evaluation, economic and health outcomes evaluated, time horizon of analysis, discounting, price and currency, method of data collection for quantity and unit cost information, cost components included, and use of sensitivity analysis. These data elements captured sufficient specifics to facilitate a detailed characterization of studies.

To evaluate study quality, validated economic evaluation checklists were employed, including the Drummond checklist [2] and Evers checklist [3]. The Drummond checklist contained 35 items evaluating the quality of an economic evaluation regarding its study design, data collection, analysis, and interpretation of results [2]. The Evers checklist included 19 items covering a core set of methodological standards for economic evaluations [3]. For each checklist, the percentage of applicable items that a study scored “Yes” was calculated. Since not all checklist items were applicable to a given study, inapplicable items were excluded when calculating the percentage of Drummond checklist and Evers checklist items that the study scored “Yes”. Higher percentage indicates better quality.

A single item classification scheme developed by Fukuda and Imanaka [8] was employed to further evaluate reporting quality. This scheme categorized each study into four levels based on transparency of their reporting: “All components of costs were described and data for both quantity and unit price of resources were reported for each component”, “All components of costs were described and data for costs in each component were reported”, “All components of costs were described but data for costs in each component were not reported”, and “Only scope of costing was described but components of costs were not described” [8].

Two researchers independently screened studies and performed data extraction. Disagreements were discussed and resolved by consensus as well as consultation with a third researcher. Descriptive statistics (e.g., frequency and percentages) were used to summarize key study design and reporting characteristics. SAS version 9.4 was used for all data analysis (SAS Institute Inc., Cary, NC).

## Results

195 studies were critically appraised. The number of studies that fully or predominantly applied the micro-costing methodology increased over time. General characteristics of the 195 micro-costing studies are summarized in Table 1. Neoplasms, infectious and parasitic diseases, and diseases of the circulatory systems were the most commonly studied disease areas, accounting for 18.5, 17.9, and 10.8% of the studies, respectively. In terms of geographic location, 36.9% were conducted in the United States, and 34.9% were performed in European countries. Most were observational studies (67.2%), while 32.8% were based on randomized controlled trials. When categorized by type of economic evaluation, a small proportion were full economic evaluations, including 24.1% cost-effectiveness analyses, 5.6% cost-utility analyses, 1.5% cost-benefit analyses, and 5.6% cost-minimization analyses. In contrast, 19.0% compared costs between two or more interventions without evaluating effectiveness and 23.1% estimated costs for a particular intervention without a comparison group or evaluating effectiveness.

**Table 1:** General characteristics of micro-costing studies

Characteristics	N (%)
Distribution of disease area (based on ICD-9-CM diagnosis chapters)^a^	
Neoplasms	36 (18.5%)
Infectious and parasitic diseases	35 (17.9%)
Diseases of the circulatory system	21 (10.8%)
Mental disorders	19 (9.7%)
Diseases of the digestive system	15 (7.7%)
Endocrine, nutritional and metabolic diseases, and immunity disorders	14 (7.2%)
Diseases of the nervous system and sense organs	7 (3.6%)
Diseases of the musculoskeletal system and connective tissue	6 (3.1%)
Diseases of the skin and subcutaneous tissue	5 (2.6%)
Injury and poisoning	5 (2.6%)
Diseases of the genitourinary system	4 (2.1%)
Congenital anomalies	4 (2.1%)
Symptoms, signs, and ill-defined conditions	4 (2.1%)
Supplementary classification of factors influencing health status and contact with health services	4 (2.1%)
Non-groupable disease/condition	3 (1.5%)
Diseases of the respiratory system	1 (0.5%)
Complications of pregnancy, childbirth, and puerperium	1 (0.5%)
Supplementary classification of external causes of injury and poisoning	1 (0.5%)
No specific disease/condition	23 (11.8%)
Distribution of study geographic location^a^	
USA	72 (36.9%)
Europe	68 (34.9%)
Asia	23 (11.8%)
Africa	19 (9.7%)
Australia/New Zealand/Micronesia	6 (3.1%)
Canada	6 (3.1%)
Latin America/Caribbean (including Mexico)	2 (1.0%)
Type of study design^a^	
Observational study	131 (67.2%)
Randomized controlled trial	64 (32.8%)
Decision analytic modeling	4 (2.1%)
Other economic modeling	2 (1.0%)
Other study design	2 (1.0%)
Type of economic evaluation^a^	
Cost effectiveness analysis	47 (24.1%)
Cost utility analysis	11 (5.6%)
Cost minimization analysis	11 (5.6%)
Cost benefit analysis	3 (1.5%)
Cost consequence analysis	12 (6.2%)
Cost comparison analysis (without evaluation of effectiveness)	37 (19.0%)
Single intervention cost analysis (without evaluation of effectiveness)	45 (23.1%)
Single intervention cost analysis (with evaluation of effectiveness)	16 (8.2%)
Cost of illness analysis	21 (10.8%)
Other economic evaluation	1 (0.5%)
Economic evaluation perspective specified	
Yes	129 (66.2%)
No	66 (33.8%)
Actual economic evaluation perspective used^a^	
Hospital/clinic/provider	111 (56.9%)
Societal	40 (20.5%)
Health care program	21 (10.8%)
Health care system	17 (8.7%)
Insurer	9 (4.6%)
Employer	2 (1.0%)
Other	15 (7.7%)
Price year specified	
Yes	131 (67.2%)
No	64 (32.8%)
Currency specified	
Yes	194 (99.5%)
No	1 (0.5%)
Inflation adjustment	
Yes	83 (42.6%)
No	7 (3.6%)
Not specified	86 (44.1%)
Not applicable	19 (9.7%)
Sensitivity analysis^a^	
Deterministic sensitivity analysis	90 (46.2%)
Stochastic (probabilistic) sensitivity analysis	13 (6.7%)
Other sensitivity analysis	6 (3.1%)
No sensitivity analysis	94 (48.2%)
Funding support^a^	
Government funding source	79 (40.5%)
Non-profit non-government funding source	47 (24.1%)
Industry sponsored study	28 (14.4%)
No funding	15 (7.7%)
Not specified	55 (28.2%)
Conflict of interest	
Yes	12 (6.2%)
No	76 (39.0%)
Not specified	107 (54.9%)

Percentages may not add to 100 due to rounding

^a^ A study may fall into more than one of the listed categories

Key design features were poorly reported (Table 1). Approximately one third of studies did not specify perspective, and only 20.5% adopted a societal perspective. 32.8% of studies did not specify price year, while 44.1% did not state whether costs from different years were inflation adjusted. 3.6% did not perform inflation adjustment when cost data were collected across multiple calendar years and did not provide justification. 48.2% of studies did not perform any sensitivity analysis to account for uncertainty in cost estimation. A large proportion of micro-costing studies did not report nature of funding (28.2%) or conflict of interest (54.9%).

Study characteristics related to cost measurement are summarized in Table 2. 49.7% referred to their own methodology as micro-costing. When assessed by the Fukuda and Imanaka [8] transparency scale, 13.8% of studies described all cost components and reported both quantity and unit cost of resources for each component, whereas 80.5% described cost components and reported data for cost in each component but did not separately report quantity and unit cost of all resources used. In terms of specific cost components estimated, virtually all studies (95.4%) included personnel costs, 88.2% included material, supplies and consumables, and 68.2% included overhead costs. Provider/staff interview, hospital administrative or cost/accounting databases, and time-motion study were the most common methods of collecting data on quantity of resource utilization, used by 36.4, 29.2 and 21.5% of studies, respectively. Invoice price, hospital administrative or cost/accounting database, and fee schedules were most commonly used to obtain unit cost data, accounting for 31.8, 30.8, and 24.1% of studies, respectively.

**Table 2:** Micro-costing related characteristics of studies

Characteristics	N (%)
Whether referred to its own methodology as micro-costing	
Yes	97 (49.7%)
No	98 (50.3%)
Separate reporting of quantity and unit cost data	
Yes	27 (13.8%)
No	168 (86.2%)
Classification of transparency of cost estimates [8]	
All components of costs were described and data for both quantity and unit cost of resources were reported for each component	27 (13.8%)
All components of costs were described and data for costs in each component were reported	157 (80.5%)
All components of costs were described but data for costs in each component were not reported	9 (4.6%)
Only scope of costing was described but components of costs were not Described	2 (1.0%)
Cost components included^a^	
Personnel	186 (95.4%)
Materials/supplies/consumables	172 (88.2%)
Overhead	133 (68.2%)
Equipment/device	118 (60.5%)
Facility	112 (57.4%)
Medication	95 (48.7%)
Transportation	74 (37.9%)
Laboratory/diagnostic/imaging test	68 (34.9%)
Productivity loss	37 (19.0%)
Food	28 (14.4%)
Furniture	22 (11.3%)
Child/elderly care	2 (1.0%)
Other	60 (30.8%)
Method of quantity data collection^a^	
Provider/staff interview	71 (36.4%)
Hospital administrative cost/accounting database	57 (29.2%)
Time-motion study	42 (21.5%)
Medical chart/record review	30 (15.4%)
Patient self-report	25 (12.8%)
Synthesis of literature	6 (3.1%)
Clinical guideline	3 (1.5%)
Other	112 (57.4%)
Not clear	32 (16.4%)
Method of unit cost data collection^a^	
Invoice price	62 (31.8%)
Hospital administrative cost/accounting database	60 (30.8%)
National/regional/provincial/hospital/insurer fee schedule	47 (24.1%)
Hospital/clinic/provider price catalogue	8 (4.1%)
Human resources/payroll record	7 (3.6%)
Other	126 (64.6%)
Not clear	37 (19.0%)

Percentages may not add to 100 due to rounding

^a^ A study may fall into more than one of the listed categories

Evaluation based on the Drummond checklist [2] and Evers checklist [3] showed moderate quality of these studies. Among the checklist items applicable to each study, the average proportion of items that scored “Yes” was 72.5% on Drummond checklist and 69.6% on Evers checklist. 72.8% did not specify analytical perspective or did not use a societal perspective and did so without justification, 83.6% did not explain why costs and benefits were not discounted, and 68.7% did not perform sufficient sensitivity analysis on important variables. While most studies measured physical units and unit costs appropriately, 14.9 and 19.5% did not provide sufficient detail to inform whether they appropriately measured physical units or valued costs, respectively.

Four studies compared the impact of micro-costing versus other costing methods, such as gross-costing, on cost estimation [9–12]. Their findings showed that, depending on services type, use of different costing methods could result in large differences in cost estimates and may affect decision-making regarding the cost-effectiveness of alternative interventions [9, 11, 12]. The micro-costing method was considered more transparent [9] and provided more detailed information to help identify cost drivers [11]. In addition, three studies compared bottom-up (i.e., assessed resource utilization for each cost item and then aggregated for a total system) and top-down (i.e., assessed resource utilization for a total system and then allocated to individual cost items) [13] costing. They demonstrated that bottom-up costing generated more accurate estimates for labor intensive services [12, 14, 15].

## Discussion

This critical appraisal of micro-costing studies in health and medicine suggests that use of micro-costing methods, while increasing, has largely varied in the quality of conducting and reporting of micro-costing analyses. Studies often did not adequately address key design issues and lacked sufficient detail or transparency in explaining study design. The few studies available that compared micro-costing with other costing methods suggest that micro-costing is the preferred method for generating accurate cost estimates, especially for studies that involve services that are labor intensive.

These findings highlight the importance and need for rigorous guidelines to help standardize the conduct and reporting of micro-costing studies. Future efforts to develop a checklist specifically tailored to micro-costing studies [7] will be instrumental. Prior checklists have evaluated full economic evaluations such as cost-effectiveness analysis and many of the checklist questions are not applicable to micro-costing studies. Moreover, many specific aspects of micro-costing studies – such as detailed enumeration of resource utilization quantity and unit costs – are not fully assessed by existing checklists. Closer attention to the design and reporting of micro-costing studies is needed from the research community, and quality standards deserve consideration by relevant professional organizations, such as future Panels on Cost-Effectiveness in Health and Medicine, the International Society for Pharmacoeconomics and Outcomes Research, and the Society for Medical Decision Making. Our study identified several limitations in current micro-costing studies, which may assist future efforts. Lack of sufficient detail about measurement of resource utilization quantity and unit costs requires particular attention in development of quality standards or checklists for micro-costing studies.

Variability existed in how studies have referred to their micro-costing method. Some studies stated bottom-up approach or did not name their method, while other studies used different terminologies such as ingredient based approach. Micro-costing methods need to be more systematically reported and consistently referred to when applied. This can further improve comparability and transparency of future studies.

This study has several limitations. First, our selection of micro-costing studies focused on those that involved systematic collection of quantity and unit costs data for a defined patient cohort. Other researchers may have used broader definitions for micro-costing and may include other types of studies such as those focusing on hospital accounting or financial interest or estimating unit costs of a particular procedure or laboratory test without a defined patient sample. Second, studies that only cited use of hospital accounting databases without providing sufficient details regarding the collection or tracking of quantity and unit cost data were excluded because lack of such information precluded verifying whether the study used micro-costing methods. As hospitals are increasingly adopting micro-costing or activity-based cost allocation systems, researchers may be able to draw on such electronic data to facilitate data collection. However, these studies should provide sufficient detail regarding key elements of micro-costing methodology in order to enhance the transparency and usefulness of information. Third, studies that claimed micro-costing but provided little information to verify their costing methodology, were excluded, thus the appraisal of included studies may overestimate the quality of existing micro-costing studies.

Although micro-costing may not always be practical, it has unique advantages compared with the more commonly used gross-costing method. It allows more accurate cost estimation, and is particularly useful for evaluating the economic impact of new interventions that lack established cost estimates [16, 17] although existing interventions, too, benefit from this methodology. Micro-costing should be the preferred method in all areas of health and medicine when such data are available. It is also increasingly a preferred method for policy purposes [18] and a measure of accountability [19]. Given the complexity of micro-costing methodology and our findings of poor quality in some key aspects of study design and reporting, more attention and effort are needed to better guide future studies. Development of a checklist specifically for micro-costing studies will be instrumental to establishing quality standards and promoting comparability and transparency in future research [7]. There is a need especially for standardizing terminology in micro-costing studies.

## Conclusion

Micro-costing studies vary widely in methodological and reporting quality, highlighting the need to standardize methods and reporting of micro-costing studies and develop tools for their evaluation.

## Supplementary Information

**Additional file 1.**

## Data Availability

All data supporting this study is provided in the results section of this paper and as supplementary information accompanying this paper.

## Abbreviations

CHEERS: Consolidated Health Economic Evaluation Reporting Standards
MEDLINE: Medical Literature Analysis and Retrieval System Online
BIOSIS Previews: Biological Abstracts, Reports, Reviews, and Meetings
